# Application of diffusion tensor imaging and blood oxygenation level-dependent magnetic resonance imaging to assess bilateral renal function induced by Iohexol in rabbits

**DOI:** 10.1186/s12882-020-01857-y

**Published:** 2020-06-03

**Authors:** Zhiqiang Wang, Hongxu Liu, Heng Meng, Duo Zhang

**Affiliations:** 1 Department of Radiology, The Affiliated Hospital of BeiHua University, 12 Jiefang Street, Jilin, 132011 P.R. China; 2 Hospital of BeiHua University, 3999 Binjiang East Road, Jilin, 132013 P.R. China

**Keywords:** Magnetic resonance blood oxygen level-dependent imaging (MR-BOLD), Diffusion tensor imaging (DTI), Contrast-induced acute kidney injury (CI-AKI)

## Abstract

**Background:**

Blood oxygenation level-dependent magnetic resonance imaging (BOLD-MRI) and diffusion tensor imaging (DTI) are useful methods for investigating the morphology and function of the kidneys, including revealing unilateral renal damage. Nevertheless, these techniques have not yet been applied for bilateral renal function. The aim of this study was to investigate whether the combination of DTI and BOLD could be used to examine different degrees of contrast-induced acute kidney injury (CI-AKI) in bilateral kidneys compared to standard methods such as serum creatinine (SCr) detection.

**Methods:**

Forty-Two New Zealand white rabbits were divided into two groups: the experimental group and the control group. Physiological saline and iodine contrast agent (iohexol, 1.0 g iodine/kg, 1.0 ml/sec) were injected via the right renal artery. DTI and BOLD-MR data were acquired longitudinally at the baseline and 1, 24, 48, and 72 h after high-pressure syringe injection to measure the apparent diffusion coefficient (ADC), fractional anisotropy (FA) and relative transverse relaxation rate (R2*). After the MR scan at each time point, three rabbits in each group were sacrificed, and changes in SCr and hypoxia-inducible factor-1α (HIF-1α) were analyzed using histopathology and immunochemistry.

**Results:**

Twenty-four hours after iohexol administration, the values of ADC and FA decreased significantly (*P* < 0.05), while R2* values increased (*P* < 0.05) in the renal cortex (CO), outer medulla (OM) and inner medulla (IM). Besides, significant negative correlations were observed among ADC, FA, and R2* in CO, OM, and IM (all *P* < 0.001, *r* = − 0.654–0.828).

**Conclusions:**

DTI and BOLD can simultaneously and non-invasively assess different degrees of CI-AKI in bilateral kidneys.

## Background

Contrast-induced acute kidney injury (CI-AKI) is the third leading cause of hospital-acquired acute kidney injury. It is defined as a sudden deterioration of renal function 1–3 days after exposure to contrast agents (serum creatinine level increased by 25% or the absolute value of serum creatinine increased by 0.5 mg/dL) [1, 2]. Nevertheless, the pathophysiological mechanism process of CI-AKI induced by different kinds of iodine contrast agents is still not completely understood. Besides, there is no consensus on preventive strategies.

SCr is commonly used as a marker to monitor the renal function impairment after administration of iodine contrast agents. Yet, SCr cannot be used to differentiate between bilateral degrees of renal function. Furthermore, when kidneys have a large reserve of renal function (impaired nephrons < 50%), no increase or a slight increase in SCr is detected [3]. Therefore, an advanced approach to the separation of bilateral renal function is of urgent importance.

MRI techniques, such as blood oxygenation level-dependent magnetic resonance imaging (BOLD-MRI) and diffusion tensor imaging (DTI), have been previously used to investigate the morphology and function of the kidneys, including revealing unilateral renal damage [4, 5]. BOLD-MRI is a functional imaging technique that monitors bilateral renal tissue deoxyhemoglobin levels. Its increasing result R2* value (transverse relaxation rate expressed as per second) indicates a higher deoxyhemoglobin concentration in renal tissue, meaning a lower oxygenation level and vice versa [6]. On the other hand, diffusion tensor imaging (DTI) that is based on the property of diffusion of water molecules in biological tissue has been increasingly used for the pathologies of nephropathy in clinic [4, 7]. DTI analyzes tissue anisotropy by analyzing the diffusion of water in different directions, thus providing information on the apparent diffusion coefficient (ADC) and the degree of directed diffusion (fractional anisotropy, FA). However, these imaging techniques have not yet been applied for distinguishing different bilateral degrees of renal function impairment after the right renal artery administration of iohexol. Thus, the aim of this study was to investigate whether the combination of DTI and BOLD could be used to examine different degrees of CI-AKI in bilateral kidneys compared to standard methods such as SCr detection.

Understanding the early pathological changes has important clinical implications for monitoring different degrees of kidney injury caused by iodine contrast agents in bilateral kidneys. In this study, we used the DTI and BOLD-MRI methods to dynamically assess the bilateral kidneys water molecule diffusion, anisotropy, and oxygenation level in CI-AKI rabbit model using an advanced 3.0 T MR scanner after iohexol injection in the right renal artery. Considering that hypoxia-inducible factor-1α (HIF-1α) is the major transcriptional regulator of hypoxia adaptation, which is significantly up-regulated and rapidly accumulated in the kidney cell nucleus under hypoxic conditions [8], bilateral kidneys histological changes and the degree of HIF-1α immunohistochemical expression were further examined to define DTI and BOLD-MRI observations.

## Methods

### Animals

Forty-Two male New Zealand white rabbits with body weight of 2.0–2.5 kg were obtained from the Department of Veterinary Medicine, BeiHua University. All the animals were housed in an environment with a temperature of 22 ± 1 °C, a relative humidity of 50 ± 1%, and a light/dark cycle of 12/12 h, and were given standard rodent chow and water *at libitum*. Besides, all animal procedures were conducted according to the National Institute of Health Guide for the Care and Use of Laboratory Animals and approved by the University of Beihua Ethics Committee (approval number 2019-Fs-06).

### Animal grouping

Under digital subtraction angiography (DSA) monitoring, all rabbits underwent the femoral artery puncture. The catheter was inserted through the guidewire into the right renal artery. The high-pressure syringe was then connected. Rabbits were then randomly divided into two groups (21 rabbits/group): the experimental group and the control group. The experimental group received iohexol (1.0 g iodine/kg, 1.0 ml/sec), while the control group was given the same volume of saline. Rabbits were anesthetized by injection of pentobarbital, 30 mg/kg via an ear vein, followed by DSA puncture, BOLD, and DTI MR scan.

### MR studies

All experiments were performed using a 3.0 T whole-body system (GE Medical Systems, Milwaukee, WI, USA), head first and supine position, along with animal coils, coronal image and BOLD-MRI sequence using an advanced multiple-echo spoiled gradient recalled echo protocol: TR = 101.5 msec; TE = 6.3–32 msec; FOV = 18 cm × 18 cm; flip angle = 30°; matrix = 160 × 160; bandwidth = 31.25 kHz; section number = 5; and section thickness = 4 mm. DTI sequence was as follows: single-shot spin-echo diffusion-weighted echo imaging, b = 0 and 1000 s/mm^2^; diffusion directions = 15; TR = 3175 msec; TE = 126 msec; FOV = 18 cm × 18 cm; matrix = 160 × 160; section number = 5; and section thickness = 4 mm. To monitor the dynamic change of intrarenal oxygenation, water molecule diffusion and anisotropy, the rabbits were scanned 1, 24, 48, and 72 h after the injection of iohexol in the right renal artery.

**Fig. 1:**
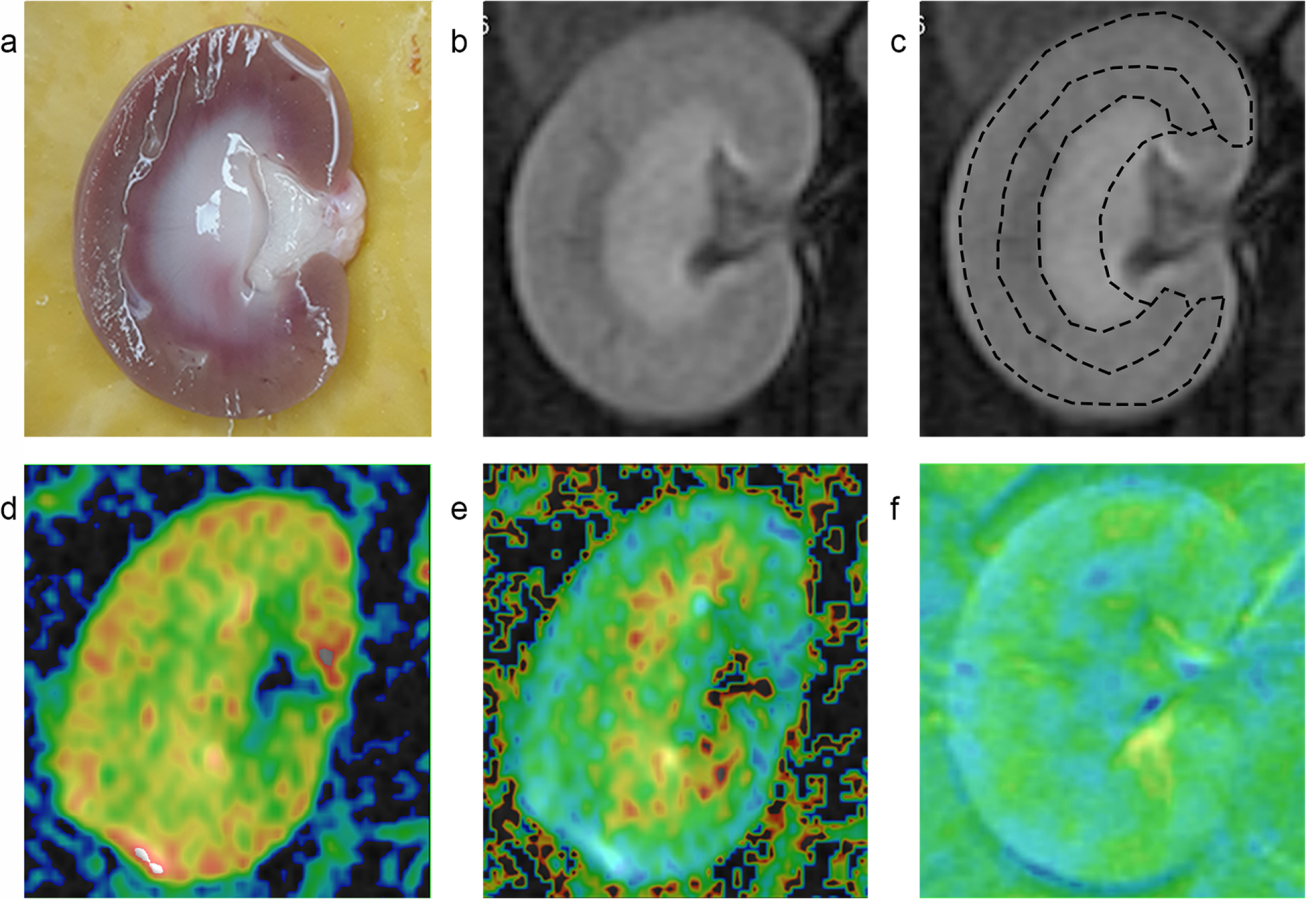
Different regions in the rabbit kidney, kidney DTI (ADC and FA) sequence parameter map, and BOLD (R2*) sequence parameter map. **a** Resected specimen. **b** MR localization map. **c** The ROIs. **d** ADC map. **e** FA map. **f** R2* map. DTI, diffusion tensor imaging. BOLD, blood oxygen level-dependent. ADC, apparent diffusion coefficient. FA: fractional anisotropy. R2*, relative spin-spin relaxation rate

Post-processing ADVANCE 4.6 Workstation software (GE Medical Systems) was used after imaging. On the localization map, a crescent-shaped region of interest was the inner medulla (IM), outer medulla (OM), and cortex (CO) from inside to outside, as shown in Fig. 1. BOLD and DTI MR data were blindly evaluated by two professional radiologists.

### Histological analysis

After the MR scan at each time point, 3 rabbits in each group were euthanized by ear vein injection of 100 mg/kg pentobarbital. Their kidneys were surgically removed, cut in half longitudinally, and the kidney capsule was removed. Samples were then fixed in 4% paraformaldehyde for 72 h. The remaining rabbits were euthanized after the experiment. Samples were then dehydrated, paraffin-embedded, cut in 5-μm thick slices, and stained with hematoxylin and eosin. Sections were blindly analyzed by a pathologist with at least 5 years of clinical experience, to assess cellular casts in the tubule lumen, lumen expansion and vacuolation of tubular epithelial cells. Scores of 0–4 were assigned to each histopathological change according to the previously published criteria [9]: 0 points, normal kidney; 1 point, mild injury (less than 5%); 2 points, moderate injury (between 5 and 25%); 3 points, severe injury (between 25 and 75%); and 4 points, severe damage (more than 75%). A mean score was calculated using 400x magnification, across five randomly selected non-overlapping areas to acquire a final renal severity score.

### HIF-1α immunohistochemical expression analysis

Sections were pretreated in the same way as described above. HIF-1α was then immunostained using the streptavidin-peroxidase technique. Briefly, after deparaffinization, antigen retrieval, and peroxidase quenching, slides were incubated with the primary antibody monoclonal anti-HIF-1α (H1alpha67; 1:200; Novus Biologicals, Littleton, CO, USA) at 4 °C overnight. Antibody binding was detected using an EliVision plus kit (EliVision™ superKIT-9922, Maixin, Fuzhou, China). Diaminobenzidine was used for staining, followed by a hematoxylin counterstain.

The HIF-1α immunohistochemical expression in the renal tubular epithelial cells was analyzed by a optical microscopy (× 400) and was scored using 0–4 points: 0 points, no or very weak nuclear staining; 1 point, < 25% of nuclear staining; 2 points, 25–50% of nuclear staining; 3 points, 50–75% of nuclear staining; 4 points, > 75% of nuclear staining [10, 11].

### Biochemistry assessment

To further verify the renal function of CI-AKI, blood SCr, blood urea nitrogen (BUN), and Cystatin C (Cys C) were measured. In each group, blood collected from the ear vein at each time point was centrifuged at the speed of 3200 rmp (4 °C) for 10 mins after the injection of iohexol or saline.

### Statistical analysis

SPSS 24.0 (Chicago, IL, USA) was used for statistical analyses, where *P* < 0.05 was considered statistically significant. Values are expressed as mean ± standard deviations. Repeated measure ANOVA and Bonferroni post-hoc test were used to compare within-group MRI parameters. One-way analysis of variance (ANOVA) and Tukey post-hoc test were used to compare intergroup MRI parameters. Spearman correlation was used to assess the relationship between R2* and ADC/FA.

## Results

DTI and BOLD-MRI data were successfully recorded. The ADC, FA, and R2* values were calculated from each time point color map.

**Fig. 2:**
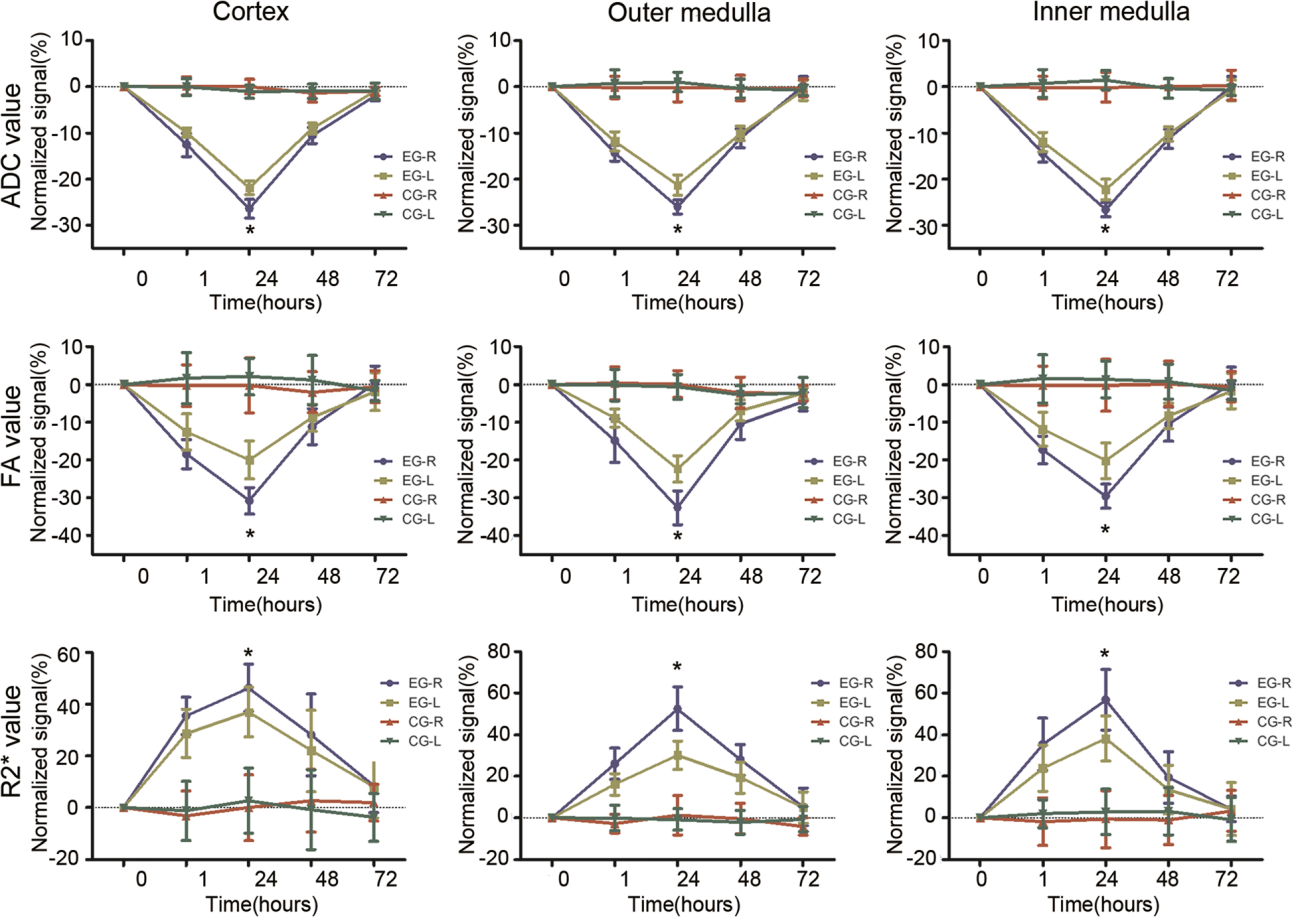
Temporal alterations in ADC, FA, and R2* levels of the bilateral renal cortex, outer medulla and inner medulla in the experimental group and control group (*n* = 6). The changes in right kidney parameters of the experimental group were most pronounced at 24 h. *P < 0.05 (EG-R vs. EG-L). One-way ANOVA Tukey *P*-value. EG-R, right renal of the experimental group. EG-L, left renal of the experimental group. CG-R, right renal of the control group. CG-L, left renal of the control group

DTI and BOLD maps can be automatically matched to the localization map, which can be used to distinguish the CO, OM, and IM in bilateral kidneys (Fig. 1). Changes of ADC, FA, and R2* values in the CO, OM, and IM before and after the injection of physiological saline and iohexol in the right renal artery are shown in Table 1 and supplementary raw data file. In the experimental group, the CO, OM, and IM of ADC and FA values at 1, 24, and 48 h after iohexol injection were low, while R2* values were elevated (all *P* < 0.05); at 72 h R2* values decreased to a level that was near to the baseline. Changes in ADC, FA, and R2* values were most evident at 24 h (Fig. 2); the changes in the right kidney parameter were more significant than the ones in the left kidney.

**Table 1:** Mean ADC (× 10^− 3^ mm^2^/s), FA and R2* (1/s) values at different time points during CI-AKI (*n* = 6)

	Baseline	1 h	24 h	48 h	72 h
ADC					
CO Right	1.66 ± 0.02	1.45 ± 0.03*	1.22 ± 0.03*	1.49 ± 0.03*	1.63 ± 0.02
Left	1.66 ± 0.02	1.49 ± 0.02*	1.29 ± 0.02*	1.51 ± 0.03*	1.64 ± 0.02
OM Right	1.59 ± 0.03	1.36 ± 0.02*	1.17 ± 0.03*	1.41 ± 0.02*	1.59 ± 0.03
Left	1.59 ± 0.02	1.40 ± 0.02*	1.25 ± 0.03*	1.43 ± 0.02*	1.58 ± 0.03
IM Right	1.57 ± 0.03	1.34 ± 0.02*	1.15 ± 0.03*	1.39 ± 0.02*	1.57 ± 0.03
Left	1.57 ± 0.02	1.38 ± 0.02*	1.22 ± 0.03*	1.41 ± 0.02*	1.56 ± 0.03
FA					
CO Right	0.39 ± 0.01	0.31 ± 0.01*	0.27 ± 0.02*	0.34 ± 0.01*	0.39 ± 0.02
Left	0.39 ± 0.01	0.34 ± 0.01*	0.31 ± 0.02*	0.35 ± 0.01*	0.38 ± 0.02
OM Right	0.41 ± 0.01	0.35 ± 0.02*	0.27 ± 0.02*	0.37 ± 0.02*	0.39 ± 0.01
Left	0.40 ± 0.01	0.37 ± 0.01*	0.31 ± 0.01*	0.38 ± 0.01*	0.39 ± 0.01
IM Right	0.49 ± 0.02	0.41 ± 0.01*	0.35 ± 0.02*	0.44 ± 0.01*	0.49 ± 0.02
Left	0.49 ± 0.02	0.44 ± 0.02*	0.39 ± 0.02*	0.45 ± 0.01*	0.49 ± 0.02
R2*					
CO Right	17.57 ± 1.09	23.78 ± 1.18*	25.68 ± 1.95*	22.41 ± 1.96*	18.98 ± 1.07
Left	17.49 ± 1.26	22.44 ± 1.05*	23.87 ± 0.83*	21.24 ± 2.02*	18.81 ± 1.36
OM Right	22.45 ± 0.95	28.25 ± 1.18*	34.16 ± 1.05*	28.67 ± 0.85*	23.64 ± 2.14
Left	22.01 ± 0.81	25.54 ± 0.90*	28.60 ± 0.87*	26.25 ± 1.19*	23.09 ± 1.77
IM Right	19.05 ± 1.25	25.68 ± 0.99*	29.71 ± 0.82*	22.65 ± 1.35*	19.84 ± 1.32
Left	18.56 ± 1.12	22.91 ± 0.70*	25.56 ± 0.76*	20.99 ± 1.29*	19.24 ± 1.38

Repeated measures ANOVA Bonferroni *P*-value and one-way ANOVA Tukey *P*-value. All data are presented as the mean ± standard deviation (SD)

*CI-AKI* contrast-induced acute kidney injury, *CO* cortex, *OM* outer medulla, *IM* inner medulla

*P < 0.05 (vs. baseline)

The ADC, FA, and R2* values of the bilateral kidneys in the control group (0.9% saline injected into the right renal artery) were determined in the same manner. As a result, these values did not significantly change, thus indicating that the changes detected in the DTI and BOLD methods were directly caused by iohexol.

**Fig. 3:**
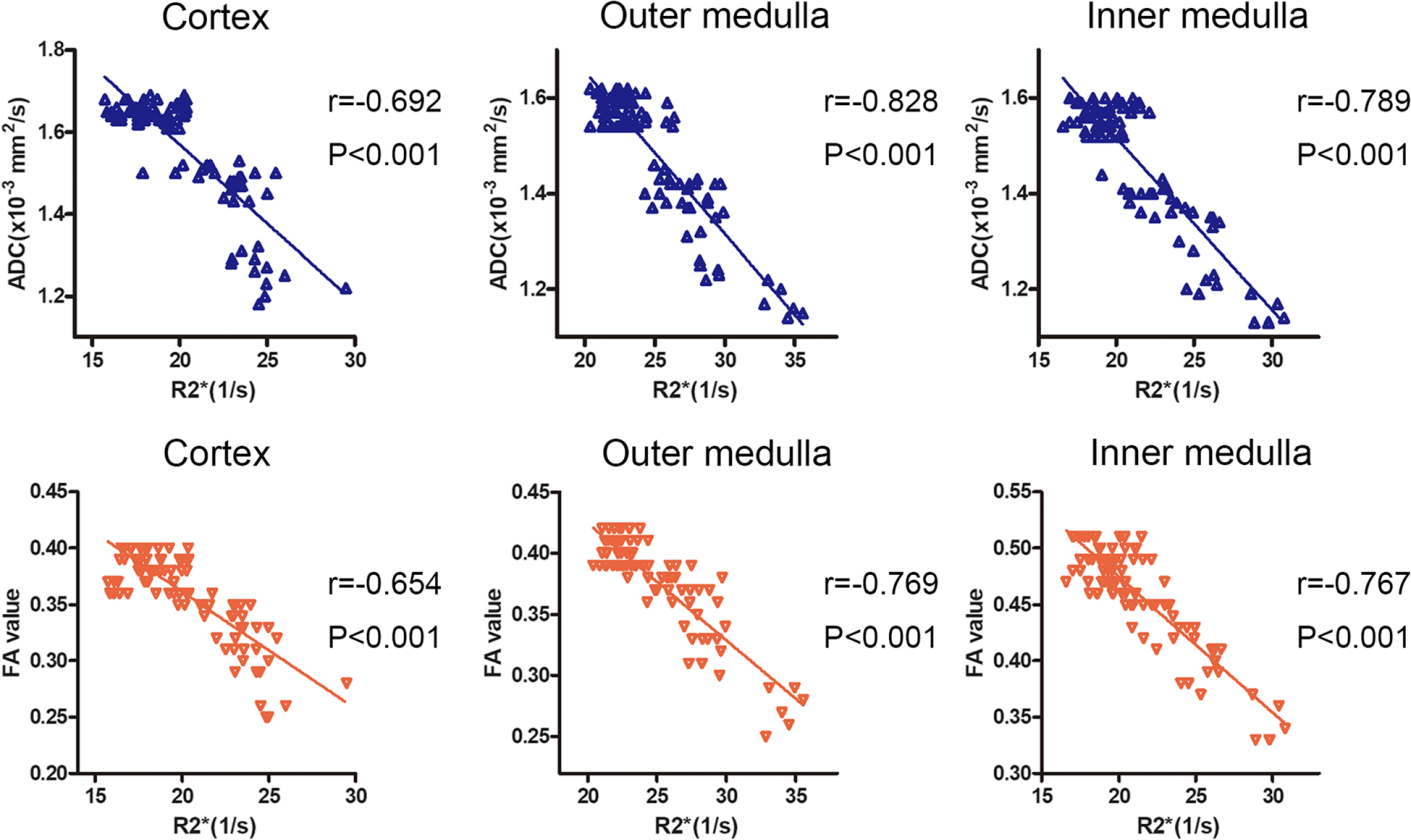
The negative correlation between R2* and ADC/FA (*n* = 6). R2* in renal CO, OM, and IM are negatively correlated with the ADC (*P* < 0.001, *r* = − 0.692; *P* < 0.001, *r* = − 0.828 and *P* < 0.001, *r* = − 0.789, respectively), FA (*P* < 0.001, *r* = − 0.654; *P* < 0.001, *r* = − 0.769 and *P* < 0.001, *r* = − 0.767, respectively). Spearman correlation analysis

In renal CO, OM, and IM, R2* values were negatively correlated with the ADC values (all *P* < 0.001, *r* = − 0.692, *r* = − 0.828 and *r* = − 0.789, respectively) and FA values (all *P* < 0.001, *r* = − 0.654, *r* = − 0.769 and *r* = − 0.767, respectively) (Fig. 3).

Blood SCr, BUN, and Cys C were measured to further verify the extent of renal injury after the injection of iohexol. The SCr concentration peaked at 48 h and was different from the baseline (*P* < 0.05), while there were no differences between the baseline at other time points (*P* > 0.05) (Table 2). Moreover, the peak change in SCr occurred one day later than that of BUN, Cys C, ADC, FA, and R2* values. The BUN and Cys C peaked at 24 h and were statistically different from the baseline (*P* < 0.05). Peak changes in cystatin C and BUN occurred at the same time as changes in ADC, FA, and R2 * values.

**Table 2:** Mean values of serum creatinine (Scr), blood urea nitrogen (BUN) and Cystatin C (Cys C) at different time points during the course of contrast-induced acute kidney injury (*n* = 3)

Time	Scr (μmol/L)	BUN (mmol/L)	Cys C (mg/L)
Baseline	36.67 ± 3.79	3.40 ± 0.36	0.57 ± 0.06
1 h	43.33 ± 3.51	3.93 ± 0.31	0.70 ± 0.10
24 h	45.00 ± 4.00	5.23 ± 0.40*	1.07 ± 0.06*
48 h	49.33 ± 5.51*	4.17 ± 0.21	0.70 ± 0.10
72 h	43.33 ± 3.06	4.07 ± 0.15	0.67 ± 0.06

One-way ANOVA and Tukey post-hoc for further comparisons

All data are presented as the mean ± standard deviation (SD)

*P < 0.05 (vs. baseline)

**Fig. 4:**
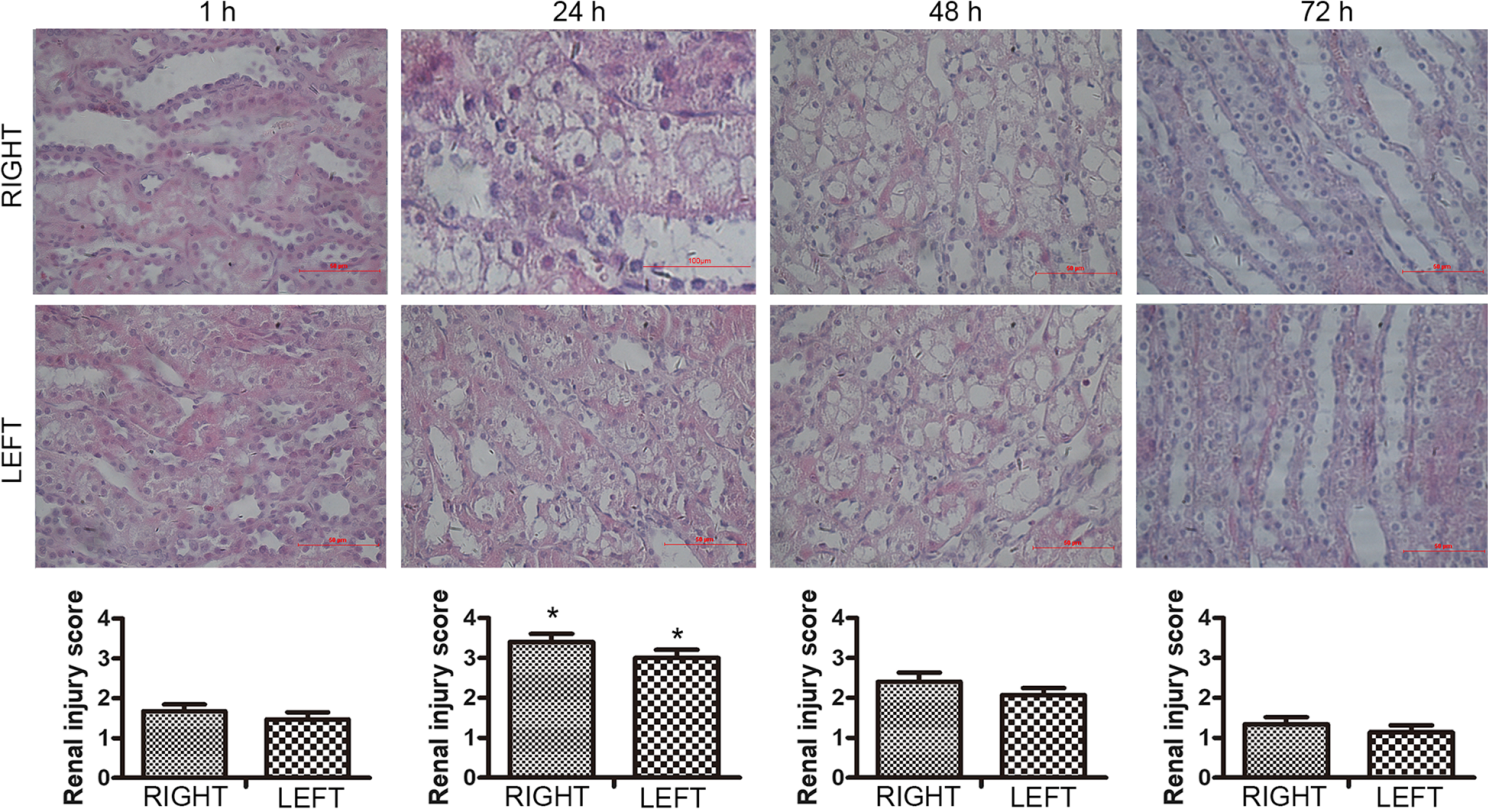
Histological effects after contrast injection via the right renal artery (HE stain, × 400)(*n* = 3). Vacuolation was primarily observed in the proximal and distal convoluted tubule epithelial cells. A small number of vacuoles were still visible in 72 h. Renal tubular lumen dilation was observed. The right renal injury score was the most severe at 24 h (*P < 0.05 vs. baseline. Baseline was normal renal tissue, and the renal injury score was 0.). Kruskal–Wallis test and pairwise comparisons

Histology was used to further verify whether MRI functional imaging DTI could assess the water diffusivity and pathological progression of CI-AKI. The H & E staining demonstrated CI-AKI progressive change in renal microscopic structure at different time points (Fig. 4): at 1 h, the renal tubular epithelial cells were slightly swollen; at 24 h, tubular epithelial cell vacuoles formed and the renal tubular lumen was dilated; at 48 h, the extent of tubular dilatation and epithelial cell vacuoles decreased; at 72 h, these changes almost disappeared. At 24 h, the extent of injury to the right kidney was slightly worse than the left kidney.

**Fig. 5:**
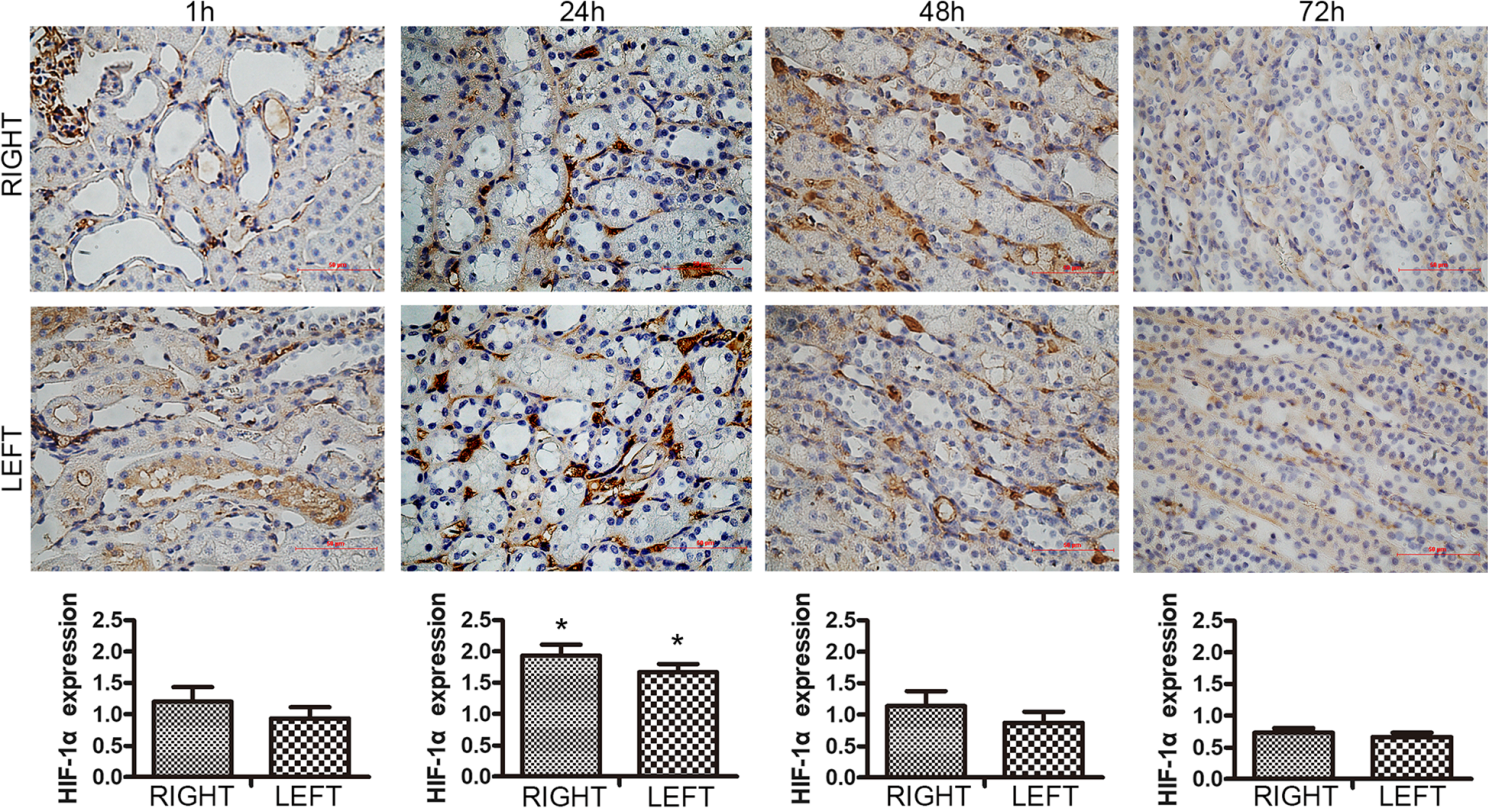
HIF-1α expression following contrast injection via the right renal artery (× 400) (*n* = 3). The right renal HIF-1α expression increased precipitously at 24 h (*P < 0.05 vs. baseline. Baseline was normal renal tissue and the HIF-1α expression score was 0.). Kruskal–Wallis test and pairwise comparisons

To verify that BOLD-MRI functional imaging can assess the oxygenation level progress of CI-AKI, the HIF-1α expression was performed after MR scanning at different time points (Fig. 5). The kidney cell nuclear staining precipitously increased 24 h after the iohexol injection, and then decreased. At 72 h, these changes basically disappeared. The right kidney HIF-1α immunoexpression was slightly higher than in the left kidney at 24 h post-injection.

## Discussion

DTI is an advanced imaging technique used to estimate the diffusion of tissue in the body by calculating the parameters of apparent diffusion coefficient (ADC) and fractional anisotropy (FA). This method provides information on the motion of water at the molecular level and information related to tissue microstructure [12]. The tubular anatomy is radially arranged, resulting in anisotropic diffusion, which can be assessed by the DTI technique [13]. In kidney diseases such as chronic kidney disease, CI-AKI, and ischemia-reperfusion injury, both ADC and FA values are significantly reduced [4, 7]. Moreover, BOLD-MRI, which can identify nephrotoxic drugs at an early stage, has been used to evaluate the effect of drugs on renal tissue oxygenation. CI-AKI patients with lower oxygenation of the kidney tissue have worse renal outcomes [6]. In this study, we found that ADC and FA values of the right kidney were significantly lower 24 h after contrast agent administration than those in the left kidney (Fig. 2). In contrast, R2* value was increased, probably because iohexol was injected via the right renal artery. These high-concentration contrast agents were directly exposed to the right kidney, causing an increase in the effect on the right kidney. In contrast, the left kidney was relatively lightly affected by blood circulation buffering. However, ADC, FA, and R2* values were not statistically different compared to the control group.

Hypoxia and direct cytotoxicity have been associated with contrast-induced kidney injury. In this study, we found that R2* value rapidly increased after iohexol injection, reaching a peak at 24 h. This may be due to the following reasons: 1) contrast agents stimulate vasoconstriction to cause hemodynamic changes and reduce renal oxygen supply [14]. 2) Iohexol is one of the nonionic monomer low-osmolar contrast media (400–800 mosmol/kg H2O), and its osmolalities are significantly higher than blood plasma (290 mosmol/kg H2O). Osmotic diuretic effects increase oxygen consumption [15]. 3) Decreased glomerular filtration rate (GFR) results in prolonged residence time of the contrast agent, increased cytotoxic edema and tubular resistance, tubular dilation, adjacent vascular compression, and further reduction of renal oxygen supply. At this time, part of the renal cell nuclear HIF-1α is markedly stained, and renal histology shows swelling and vacuolation of renal tubular epithelial cells. The swelling of the renal tubular epithelial cells and the increase of vacuoles cause changes in intracellular water, which limits the diffusion of renal water and decreases the ADC value [16]. Tubular expansion, cellular debris, reduced tubular flow velocity, cellular casts in the tubule lumen, and tubular damage may impair directional diffusion and restrict FA [17]. Subsequent R2*, ADC, and FA values gradually returned to the baseline level, and this trend was related to the metabolism of iodine contrast agent in vivo.

Previous studies have stated that 99% of the iodine contrast agent is eliminated through urine within 24 h of injection [18]. Histological changes and HIF-1α stained changes are also gradually recovered. Interestingly, our study showed that the time point of BUN, Cys C, R2*, ADC, FA values recovery occurred 1 day earlier than SCr. This may be because SCr is affected by several factors, such as age, weight, muscle mass, and various medications, which slow down the rate of change [19, 20]. Longitudinal dynamic studies have illustrated that R2*, ADC, and FA are extremely sensitive to simultaneous monitoring of different degrees of pathological changes in both kidneys during the CI-AKI process. In this study, DTI was used to analyze tissue anisotropy by examining the diffusion of water in different directions, while BOLD-MRI was used to measure renal tissue deoxyhemoglobin levels. A combination of these methods provided independent diagnostic information on kidney function, and thus can be used to evaluate CI-AKI pathophysiological process more accurately.

The research of the effects of iohexol on renal water diffusion, anisotropy, and oxygenation is essential to comprehensively understand the mechanism of CI-AKI and prevent the progress of renal hypoxia. DTI and BOLD-MRI functional imaging provides an advanced method for dynamic non-invasive monitoring of kidney injury in most clinical common kidney diseases, including CI-AKI, chronic kidney disease, renal artery stenosis, and ureteral obstruction [4, 13, 21]. Our data suggested that these techniques could simultaneously compare bilateral renal function and monitor the treatment response to common renal hypoxic diseases. Yet, to achieve reproducibility in animals and humans, normalization of measured data is essential. In our experiments, the normalized calculation method is the percentage change of parameters at different time points relative to the baseline.

The rabbit model is a suitable alternative to study kidney changes in CI-AKI because it can be obtained by injecting a contrast agent only once in healthy rabbits, so there are no other interfering factors compared to clinical studies [22, 23]. Moreover, rabbits have larger kidneys (better spatial resolution and signal-to-noise ratio at 3.0 T MRI), and their protein sequence is more closely related to humans compared to mice and rats [24]. Besides, in this study, the rabbit CI-AKI model was established using a renal artery, which is very similar to the procedure of clinical selective renal angiography.

Based on BUN, Cys C, and histological examination, CI-AKI was established 24 h after contrast agent injection. At this time, the ADC and FA values were 24.1 and 25.9% lower than the baseline, respectively, and the R2 * values were 43.5% higher than the baseline. These data further suggested that DTI and BOLD could serve as a novel biomarker for the early detection of AKI. Medical staff can objectively decide who among angiographic patients can be safely discharged after 24 h and who should be retained for a further 24 h when MRI parameters change should be evident in those developing CI-AKI. We believe that MRI parameters are more clinically relevant, practical, and non-invasive. In our future studies, we plan to expand the sample size and further study the range of reference values of DTI and BOLD parameters in CI-AKI.

Our study has certain limitations. Firstly, the ADC, FA, and R2* values of CO, OM, and IM were manually sketched based on the localization map. In the measurement, a large crescent-shaped ROI drawing method was used to include the CO, OM and IM to the greatest extent to improve the sensitivity and objectivity by optimizing the measured tissue envelope, and thus reduce deviations [25]. Secondly, BOLD-MR cannot distinguish whether the hypoxia during CI-AKI was caused by decrease in oxygen supply or an increase in oxygen consumption. Lastly, due to the poor time efficiency of respiratory-triggered methods, DTI and BOLD scanning were performed with a free-breathing method.

## Conclusions

DTI and BOLD MRI can be used for analyzing renal water diffusion, anisotropy, and oxygenation. Our study showed that the level of renal oxygenation was significantly reduced, diffusion and anisotropy were decreased, and the injury of the right kidney was worse than the left kidney after the injection of iohexol in the right kidney, which suggests a potential application of DTI and BOLD MRI for non-invasive assessment of bilateral renal pathophysiological process in CI-AKI.

## Supplementary information

**Additional file 1.** Raw data of R2*, ADC and FA values of bilateral renal cortex, outer medulla and inner medulla (*n* = 6).

## Data Availability

All data generated or analyzed during this study are included in this published article and its supplementary information raw data file.

## Abbreviations

DTI: Diffusion tensor imaging
BOLD: Blood oxygenation level-dependent
MRI: Magnetic resonance imaging
ADC: Apparent diffusion coefficient
FA: Fractional anisotropy (R2*)
R2*: Relative transverse relaxation rate
HIF-1α: Hypoxia-inducible factor-1α
CO: Cortex
OM: Outer medulla
IM: Inner medulla
CIAKI: Contrast-induced acute kidney injury
SCr: Serum creatinine
DSA: Digital subtraction angiography
BUN: Blood urea nitrogen
Cys C: Cystatin C
